# Assessing the Feasibility of a Web-Based Weight Loss Intervention for Low-Income Women of Reproductive Age: A Pilot Study

**DOI:** 10.2196/resprot.4865

**Published:** 2016-02-26

**Authors:** David N Cavallo, Jessica A Sisneros, Ashley A Ronay, Cheryl L Robbins, Stephanie B Jilcott Pitts, Thomas C Keyserling, Ai Ni, John Morrow, Maihan B Vu, Larry F Johnston, Carmen D Samuel-Hodge

**Affiliations:** ^1^ Case Western Reserve University Department of Nutrition Cleveland, OH United States; ^2^ UNC Center for Health Promotion and Disease Prevention Chapel Hill, NC United States; ^3^ East Carolina University Brody School of Medicine Department of Public Health Greenville, NC United States; ^4^ Centers for Disease Control and Prevention National Center for Chronic Disease Prevention and Health Promotion Division of Reproductive Health Atlanta, GA United States; ^5^ East Carolina University, Brody School of Medicine Department of Public Health Greenville, NC United States; ^6^ UNC Center for Health Promotion and Disease Prevention Department of Medicine UNC School of Medicine Chapel Hill, NC United States; ^7^ University of North Carolina at Chapel Hill Department of Biostatistics Chapel Hill, NC United States; ^8^ Pitt County Health Department Greenville, NC United States; ^9^ UNC Center for Health Promotion and Disease Prevention UNC Gillings School of Global Public Health and School of Medicine Chapel Hill, NC United States

**Keywords:** Obesity, Nutrition, Physical Activity, Minority Health, Healthcare Disparities, Intervention Studies, Internet, women, weight loss, mhealth

## Abstract

**Background:**

Low-income women of reproductive age are at increased risk for obesity and resulting increases in the risk of maternal/fetal complications and mortality and morbidity. Very few weight-loss interventions, however, have been targeted to this high-risk group. Based on the high prevalence of social media use among young and low-income individuals and previous successes using group formats for weight-loss interventions, the use of social media as a platform for weight-loss intervention delivery may benefit low-income women of reproductive age.

**Objective:**

Examine the feasibility of delivering group-based weight-loss interventions to low-income women of reproductive age using face-to-face meetings and Web-based modalities including social media.

**Methods:**

Participants attended a family planning clinic in eastern North Carolina and received a 5-month, group- and Web-based, face-to-face weight-loss intervention. Measures were assessed at baseline and 20 weeks.

**Results:**

Forty participants enrolled, including 29 (73%) African American women. The mean body mass index of enrollees was 39 kg/m^2^. Among the 12 women who completed follow-up, mean weight change was -1.3 kg. Participation in the intervention was modest and retention at 5 months was 30%. Returnees suggested sending reminders to improve participation and adding activities to increase familiarity among participants.

**Conclusions:**

Engagement with the intervention was limited and attrition was high. Additional formative work on the barriers and facilitators to participation may improve the intervention’s feasibility with low-income women of reproductive age.

## Introduction

Low-income women of reproductive age (WRA) are at increased risk for obesity [1], which is in turn associated with increased risk of maternal and fetal complications [2] during pregnancy, and morbidity [3] and mortality [4] throughout life. Few weight-loss intervention studies are targeted to low-income women [5]. Findings from recently reviewed individual- and group-format weight-loss interventions are encouraging, particularly group-based interventions [5]. To our knowledge, there is a dearth of studies investigating Web-based weight-loss interventions including social media as a mechanism to encourage social support and healthy behavior changes among low-income WRA.

Web-based delivery of intervention components may reduce barriers to intervention participation experienced by low-income WRA (e.g., lack of time or support) [6]. For example, social media websites (e.g., Facebook) may be effective social support platforms for weight-loss interventions among this population. These websites are used by 84% of Internet users aged 18-29 years, are more likely to be used by lower income Internet users, and offer group communications tools and the capacity for establishing networks of online friends [7]. Given these features, social media websites are potentially effective delivery mechanisms for group-based peer support interventions [8].

Although there is a growing body of research examining Web-based weight-loss interventions, [9] few have targeted low-income WRA and, to our knowledge, none have used social media with this group. The purpose of the current study was to examine the feasibility of delivering a previously tested group-based weight-loss intervention [10] adapted to low-income WRA using Web-based educational content and social media.

## Methods

### Study Design

This feasibility study employed a single group pre-post design. We piloted a weight-loss intervention among female participants enrolled from a county health department family planning clinic and assessed outcome measures at baseline and 5 months.

### Recruitment

The Integrated Screening and Health Assessment, Prevention, and Evaluation (InShape) Study [11] screened 462 participants ages 18-44 years for cardiovascular disease risk factors. InShape eligibility criteria were being nonpregnant, English speaking, and attending an initial or annual family planning clinic visit at a health department located in eastern North Carolina. Each participant was given a brochure with information about enrolling in a weight-loss study or lifestyle study. Women who had a BMI greater than 27.5 kg/m^2^ were considered eligible for the weight-loss study. Although the United States Preventive Services Task Force recommends that patients with a BMI greater than 30 take part in weight-loss interventions [12], those in the higher half of the overweight category (27.5 to 29.9) often wish to lose weight for both health and aesthetic reasons, and thus were invited to be in this study.

Participants who expressed interest in the weight-loss study were contacted by phone and invited to an enrollment visit. To have sufficient numbers for the group format component of the intervention, the intervention did not begin until all participants were enrolled. The Institutional Review Board of the University of North Carolina at Chapel Hill approved and monitored this study.

### Program Adaptation

The InShape weight-loss intervention content was based on the Weight-Wise program, a group-based, 16-session, behavioral weight-loss intervention targeting low-income, midlife (40-64 years old) women [10]. The Weight-Wise program emphasizes goal setting, self-monitoring, feedback, and education to promote dietary and physical activity (PA) recommendations adapted from the Diabetes Prevention Program [13]. For this study, the Weight-Wise program was modified to be more feasible by maintaining the key behavioral components, decreasing the number of face-to-face sessions from 16 to 5, adapting content originally covered in the group sessions for delivery through Web-based modules, and including a social media component for social support.

### Enrollment Visit

**Figure 1:**
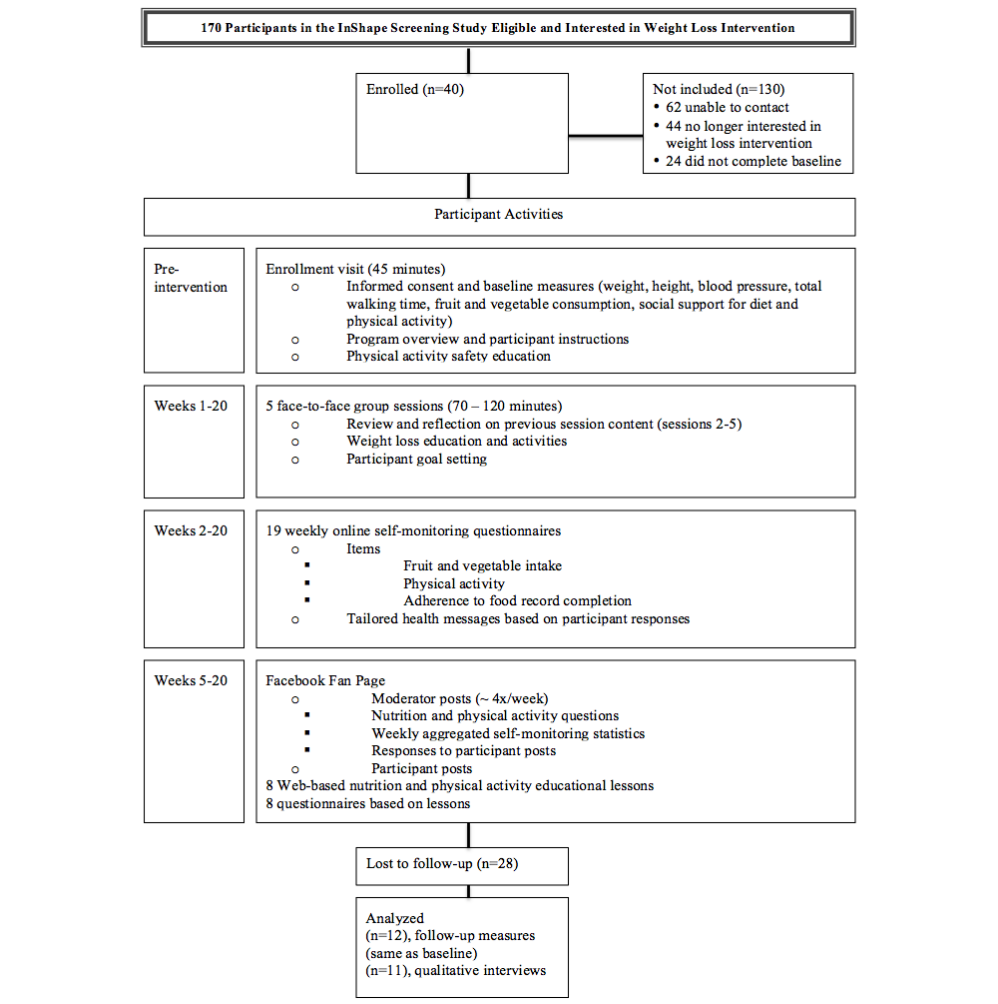
Study flow diagram of participant activities.

At the enrollment visit, conducted at the study research office, the interventionist first obtained informed consent and completed baseline measures (Figure 1). Then, during a 30 minute face-to-face, enrollment and counseling visit, the interventionist provided a program overview, instructed participants on how to perform the research study self-monitoring, provided PA safety information, and asked participants to set behavioral goals. A body weight scale, calorie counting book, and pedometer were provided to participants to take home.

### Group Sessions

Once a sufficient number of participants completed the enrollment and counseling visit, the remaining intervention activities were initiated. Five face-to-face group sessions (70-120 minutes) were held over a 20-week period at the health department on days and times that participants indicated were convenient (each session was offered 2 times during the week). The mean interval between the enrollment visit and the first group session was 85 (6-148) days. During each session, participants reflected on previous session content; received new intervention content, including hands-on activities such as PA or food preparation demonstrations; and completed an action plan for the coming week’s goals.

### Facebook Fan Page

**Figure 2:**
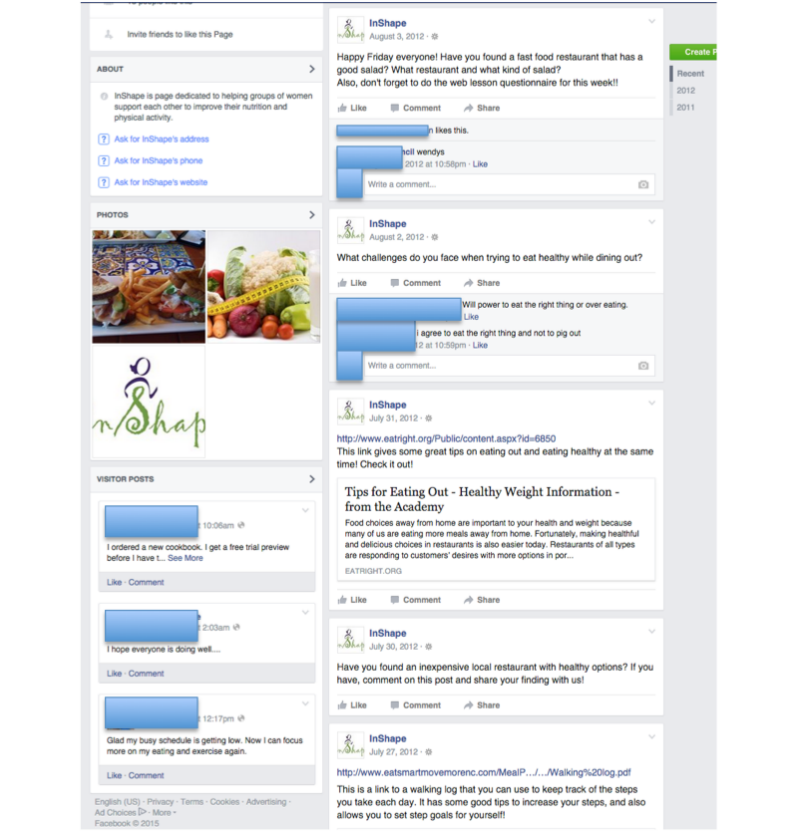
InShape Facebook Fan Page.

At the onset of intervention activities, participants were invited to “like” a Facebook fan page created for the study, where participants could model healthy behaviors and support others in their dietary and PA behavior changes (Figure 2). Study staff “seeded” the fan page with weekly remarks designed to encourage participation, including ice breaker questions, questions related to lesson and group session content, and feedback about aggregate data from participants’ weekly self-monitoring.

### Web-Based Educational Lessons

**Figure 3:**
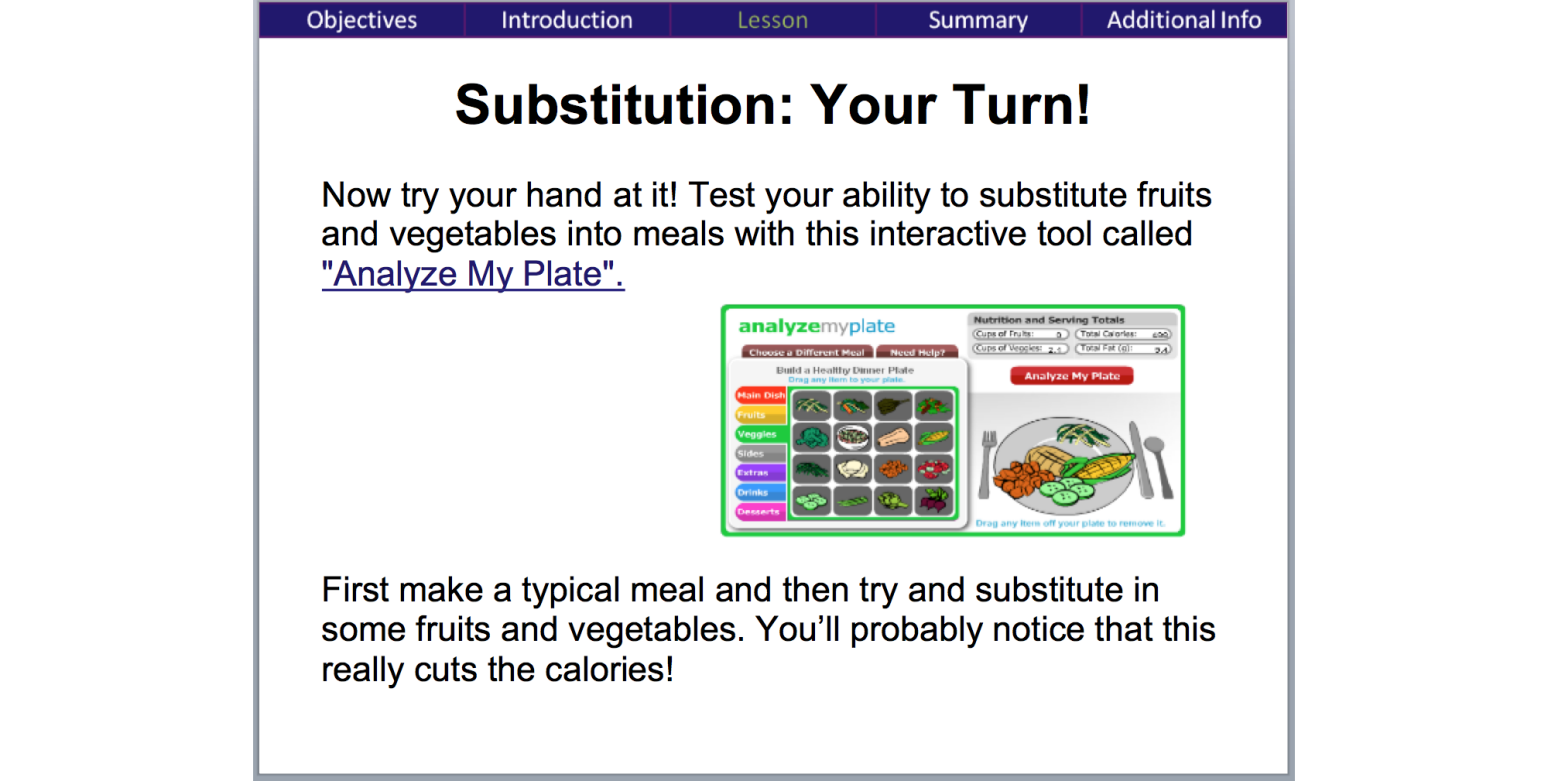
InShape Web-based educational lesson.

Using Google docs, a Web-based document sharing program, we provided participants with hyperlinks (via email) to 8 Web-based nutrition and PA educational lessons every 2 weeks for 16 weeks, beginning 1 month after intervention activities were initiated (Figure 3). Each lesson was followed by an email invitation to complete a Web-based lesson questionnaire. These questionnaires asked participants about the topics covered in the Web-based lessons and provided reinforcing educational messages following each question. Lessons were independent (i.e., participants were not required to complete earlier lessons to participate in subsequent lessons).

### Self-Monitoring with Tailored Feedback

The interventionist sent participants weekly emails containing a link to a brief, 6-question online self-monitoring questionnaire where participants were asked to self-report weight, frequency of keeping a food record, fruit and vegetable intake, amount of moderate physical activity, and number of steps. Tailored tips for improving or maintaining healthy behaviors were delivered to participants based on their questionnaire responses.

### Qualitative Interviews

After completion of the intervention, we conducted 15-minute in-person and telephone qualitative interviews to assess intervention acceptability and barriers to participation. Incentives ($20 gift cards) were given at measurement visits (baseline and 5 months) and upon completion of qualitative interviews, and also were available based on “InShape points,” which were earned by participating in intervention activities. We conducted 5 monthly drawings for points-based incentives. The number of entries/participant equaled the number of their InShape accumulated points.

### Measures

Trained personnel collected data at baseline and 5 months. Weight and height (baseline visit only) were measured twice without shoes using an electronic scale and portable stadiometer and then averaged. Blood pressure was measured 3 times (after the participant was seated for 5 minutes and then at 1 minute intervals) with an Omron HEM-907XL automated blood pressure monitor and averaged.

We assessed multiple other outcomes through self-report at baseline and 5 months. Total walking time was documented with the RESIDE PA survey [14] and daily fruit and vegetable servings with a Block Rapid Food Screener [15]. Social support for diet and PA were measured using the Social Support and Eating Habits/Exercise scales [16]. Self-efficacy for diet and PA behavior change were each measured with a single question (eg, “On a scale of 1 to 5, how sure are you that you can walk or do a similar activity for 30 minutes or more on 5 or more days per week?”).

Using the Qualtrics survey program (Qualtrics, Provo, UT) we recorded the frequency of participants’ use of the online self-monitoring questionnaires and completion of the Web-based lesson questionnaires. The interventionist manually recorded Facebook fan page contributions and logged participant attendance at group sessions. At the end of the intervention period, participants also completed an acceptability questionnaire and a qualitative interview to solicit their thoughts about and experiences with the intervention and suggestions for improving it.

### Statistical Analysis

Descriptive statistics were used to describe baseline characteristics and the frequency of participant involvement in intervention activities. Due to the limited sample size at 5 months, we did not use statistical tests to examine differences in measures at baseline and 5 months. Statistical analysis was performed with SAS software (Version 9.3, Cary, NC). Analysis of qualitative interviews included identifying key themes and quantifying instances of specific responses.

## Results

### Recruitment

Of the 251 (54%) participants in the InShape Screening Study who were eligible for the weight-loss intervention based on a BMI greater than 27.5 kg/m^2^, 51 (20%) expressed an interest in the weight-loss study and 119 (47%) in either the lifestyle or the weight-loss study. Of these 170 eligible and initially interested participants, we successfully contacted 108 (64%). Of those contacted, 64 (59%) expressed continued interest in the weight-loss study with 40 (63%) completing the baseline visit to comprise the study sample.

### Participant Characteristics

The majority of participants (N=40) were under 30 years of age, African American, low-income, and uninsured (Table 1). One-quarter of participants reported high blood pressure or had a measured systolic higher than 140 mmHg or diastolic greater than 90 mmHg and the average BMI was 39 kg/m^2^. Over half were categorized as having class II or III (extreme) obesity. Most participants reported using the Internet (95%) and Facebook (83%).

**Table 1:** Baseline characteristics of female participants age 18-44 years (N=40).

Characteristic		n (%) or mean (SD)
Age, y, mean (SD)		30 (6.5)
**Race** ^a^		
	African American	29 (73)
	White	10 (25)
**Ethnicity** ^b^		
	Hispanic	5 (13)
Total annual household income		
	< $20,000	34 (85)
	$20,000-$39,999	5 (13)
	$40,000-$69,999	1 (3)
Currently employed full time		16 (40)
**Health insurance**		
	Private	2 (5)
	Medicaid	7 (18)
	No insurance	31 (78)
**Education**		
	Some high school	7 (18)
	High school graduate	19 (48)
	At least some college	14 (35)
Weight, kg, mean (SD)		106.0 (25.8)
**BMI, kg/m** ^**2**^ **, mean (SD)**		39 (8.5)
	Overweight (BMI 25-29.9)	7 (17)
	Class I Obesity (BMI 30-34.9)	8 (20)
	Class II Obesity (BMI 35-39.9)	8 (20)
	Class III Obesity (BMI 40+)	17 (42)
High blood pressure (patient self-report, systolic ≥ 140 mmHg, or diastolic ≥ 90 mmHg)		10 (25)
High blood cholesterol (self-report)		4 (10)
Current smoker (self-report)		12 (30)
Diabetes (patient self-report of diagnosis or A1cValue ≥ 6.5)		4 (10)
Fruit and vegetables, servings/day, mean (SD)		3.3 (1.7)
Minutes walked, weekly, mean (SD)		59.1 (174.5)
Technology use		
	Use Internet	38 (95)
	Use Facebook	33 (83)

^a^Data for 1 participant were missing.

^b^Two participants did not report ethnicity.

### Participant Outcomes

Twelve participants (30%) completed 5-month measures (study returnees). At the end of the intervention period (5 months), the difference in mean weight among the 12 study returnees was -1.3 kg (SD=4.4 kg) with a range in weight change of -9 to 5 kg. Of note, 7 of 12 participants lost weight. Mean change in walking time was 116.3 minutes/week (SD=191.6 minutes/week) with a range of 0 to 675 minutes/week. Mean change in servings/day of fruit and vegetables was 0.5 servings/day (SD=1.5 servings/day) with a range of -2 to 3 servings/day.

### Participant Use

**Figure 4:**
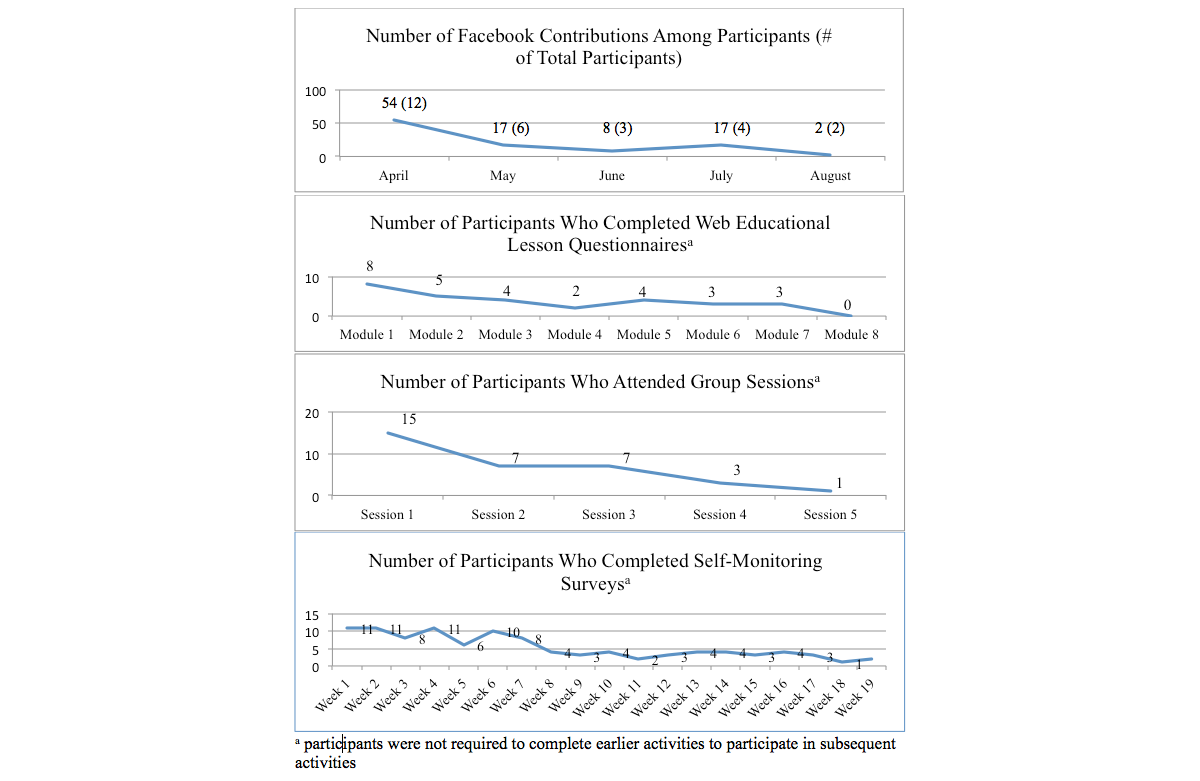
Participation in intervention activities (N=40).

Intervention activity participation was modest at the beginning and declined over time (Figure 4). A total of 12 participants contributed 98 comments to the Facebook group (mean=8, range = 1-20). Participants exchanged a variety of social support messages (eg, “Hi ladies I am looking for a walking partner I live in [City]” [response] “I live too far for an everyday thing...but we could always meet at a park! Lemme know!”) Completion of Web lesson questionnaires declined from 8 women (module 1) to none (module 8). Eighteen women participated in 1 or more face-to face group sessions. Completion of self-monitoring questionnaires declined from 11 (week 1) to 1 (week 18).

### Participant Evaluation of Intervention

Qualitative analysis (n=11) suggested that participants had hectic lives and work schedules that interfered with intervention participation and behavior change. Some returnees reported lack of access to the Internet, lack of transportation, and a desire for additional tools like digital calorie counting applications and food scales. Returnees wanted more frequent group sessions and more meeting times and suggested more text and email reminders. Suggestions for improving the Facebook component of the program included having team building exercises, enlisting friends, and providing a list of participants to each person to facilitate more face-to-face interaction with other participants. Participants also suggested more moderator posts, reminders, and greater frequency of providing incentives.

## Discussion

Despite the need for weight loss among our sample of overweight and obese participants, only a small percentage were interested in joining the study and 30% of participants completed the intervention. On average, minimal changes in weight were observed among returnees.

### Principal Findings

Use of Web-based components was low and eroded over time. Despite the high levels of self-reported Internet and social media use, it is possible that participants’ Internet access was episodic rather than continuous, which may have hindered participation in Web-based components. Lack of familiarity with other participants prior to using the Facebook fan page may have limited engagement in the social media intervention component. Attrition in the current study was greater than previous Web-based weight-loss intervention studies that mostly included older populations, including some that used Facebook for intervention delivery [17,18]. Participation was low for group sessions as well, indicating that there are likely reasons unrelated to mode of delivery that affected engaging in the intervention. The long interval between expressing interest and beginning study activities (mean 85 days) may have exacerbated traditional barriers to participation that arise over time (eg, moving frequently). This delay was based on our desire to have a “critical mass” of participants for the social media component at the outset and to have the components delivered in concert.

Future social media interventions should plan for this contingency by having rapid recruitment procedures in place. It may also be necessary to provide some intervention components or other content to participants in this lag period to maintain interest. In addition, having multiple intervention components on different platforms (ie, Facebook, Google, email, and Qualtrics) may have produced too much burden for participants. Retention could possibly be improved by delivering all intervention components through 1 platform. Other factors endemic to our study population may have also played a role in limited engagement and high attrition. For instance, young adults cite different weight loss motivations (eg, appearance and social pressure) compared to older adults (eg, health concerns) [19]. Our emphasis on the health benefits of weight loss may not have resonated with young women and, given nearly 75% of the sample was African American, greater normative acceptance of higher BMI may have limited the effectiveness of our intervention [20].

Several suggestions for improving levels of engagement in future studies targeting low-income WRA can be drawn from our results. Based on participant feedback about the need for reminders and the high penetration of cellular phones in low-income populations, [21] incorporating text message reminders or content could improve participation. Our qualitative data also suggest that social media-based interventions should build familiarity between participants prior to intervention activities. This could be accomplished by establishing stronger ties between intervention participants or incorporating existing online social ties (eg, friends, family) into social media interventions. Screening for participant motivation, which was not done in this study, has been undertaken in previous weight-loss intervention studies that reported greater participation and retention [10,22]. Finally, weight-loss intervention content should be tailored to factors that are motivating for young adults, such as physical appearance and social factors [19].

### Limitations

This study has several limitations. There was a significant lag between many participants expressing interest and beginning the intervention. Given the small number of returnees, we did not undertake statistical testing. Misclassification is possible since we measured PA by self-report, which is subject to overreporting, [23] and our measure of Internet access did not differentiate between episodic and continuous access. Nonreturnees, who may have had less positive attitudes toward the intervention or expressed additional barriers to participation, were not included in qualitative interviews. We recommend that future intervention research targeting populations with traditionally high attrition develop a priori strategies for collecting data from nonreturnees who may be in the best position to explain the most salient factors associated with their lack of participation.

### Conclusions

Although use of the Web and social media in particular is prevalent among young, lower income women, participation in the Web-based components of our intervention was limited. Additional formative research with larger samples of low-income WRA should be conducted in order to develop novel intervention strategies for weight loss among low-income women of reproductive age who are at high risk for both chronic disease and poor pregnancy outcomes.

## Abbreviations

InShape: Integrated Screening and Health Assessment, Prevention, and Evaluation
PA: physical activity
WRA: women of reproductive age
